# Contrasting Patterns of Genetic Diversity and Divergence Between Landlocked and Migratory Populations of Fish *Galaxias maculatus*, Evaluated Through Mitochondrial DNA Sequencing and Nuclear DNA Microsatellites

**DOI:** 10.3389/fgene.2022.854362

**Published:** 2022-05-19

**Authors:** Marcela P. Astorga, Andrea Valenzuela, Nicolás I. Segovia, Elie Poulin, Luis Vargas-Chacoff, Claudio A. González-Wevar

**Affiliations:** ^1^ Instituto de Acuicultura, Universidad Austral de Chile, Puerto Montt, Chile; ^2^ Departamento de Biología Marina, Facultad de Ciencias del Mar, Universidad Católica Del Norte, Coquimbo, Chile; ^3^ Instituto Milenio Biodiversidad de Ecosistemas Antárticos y Sub-Antárticos (BASE), Santiago, Chile; ^4^ Departamento de Ciencias Ecológicas, Facultad de Ciencias, Universidad de Chile, Santiago, Chile; ^5^ Instituto de Ciencias Marinas y Limnológicas (ICML), Facultad de Ciencias, Universidad Austral de Chile, Valdivia, Chile; ^6^ Centro FONDAP de Investigaciones en Dinámica de Ecosistemas Marinos de Altas Latitudes (IDEAL), Universidad Austral de Chile, Valdivia, Chile

**Keywords:** mtDNA, Chile, estuary, lake, last glacial maximum

## Abstract

*Galaxias* species are interesting biogeographic models due to their distribution and different types of life cycles, with migratory and landlocked populations. To obtain a better understanding of the genetic consequences of the Quaternary glacial cycles in *Galaxias maculatus*, in this work we compared landlocked and migratory populations collected in areas that were differentially affected by ice advances and retreats. We included nine populations of *G. maculatus*, four collected from lakes (landlocked) and five from their associated estuaries/rivers (migratory) in three estuary-lake systems across southern Chile. Genetic analyses were performed using the mitochondrial control region and nine microsatellite loci. Genetic diversity measured with both markers was significantly higher in migratory than in landlocked populations across the study area. The levels of genetic differentiation showed higher differentiation among lakes than estuaries. Genetic diversity was higher in migratory populations located in areas that were less impacted by ice during Quaternary glacial processes. These results may be the consequence of recent recolonization of small freshwater bodies following the Last Glacial Maximum (LGM). Finally, the greatest differentiation was observed in populations that were exposed to continental ice advances and retreats during the LGM. Thus, in the present work we corroborate a pattern of differentiation between lakes and estuaries, using mtDNA sequences and microsatellite nuclear markers. This pattern may be due to a combination of biological factors, i.e., resident non-migratory behaviour or landlocking and natal homing-in, as well as geological factors, i.e., Expansion-Contraction Quaternary glacial biogeographic processes.

## 1 Introduction

*Galaxias maculatus* is one of the freshwater fish species with the broadest distribution in cold and temperate regions of the Southern Hemisphere, including populations in southern Australia, Tasmania, New Zealand, South America, and the Falkland/Malvinas Islands (McDowall, 1970; Dyer, 2000; Habit et al., 2006). This species presents amphidromous behaviour, i.e., it reproduces in lakes and rivers and the larval stages migrate to the sea, where the individual develops before returning to complete its growth in freshwater ecosystems (McDowall, 1997). In addition, the presence of resident or landlocked populations has been observed in lakes and rivers, as well as migratory populations that maintain amphidromous behaviour (Delgado et al., 2019). This species is therefore considered an interesting model for historical biogeographic studies due to its particular distribution (Waters and Burridge, 1999; Waters et al., 2000; Zattara et al., 2005; Burridge et al., 2012; Waters et al., 2020), its response to glacial events (Zemlak et al., 2010; Zemlak et al., 2011; Carrea et al., 2012, 2013; González-Wevar et al., 2015a; González-Wevar et al., 2015b; Victoriano et al., 2020), and its migratory and non-migratory behaviour in the same habitat (Delgado et al., 2019; Delgado and Ruzzante, 2020). This variability makes *G. maculatus* a suitable model for evaluating genetic indicators associated with these unique characteristics, as this species has been considered to represent an intermediate evolutionary step between migratory behaviours of marine and freshwater organisms (Corush, 2019).

The glacial history of southern South America is relatively well understood. Continental ice sheet advances and retreats during the Quaternary occurred across the Pacific margin of Patagonia and generated major shifts in sea level, climate and landscape (Hulton et al., 2002; Rabassa et al., 2005, 2011; Hein et al., 2010). During the Last Glacial Maximum the Patagonian Ice Sheet expanded to an area stretching from 35°S to 56°S, and covered most of the Pacific fjords and channels of Patagonia (Clapperton, 1994; McCulloch et al., 2000). Accordingly, the Quaternary geomorphology of Patagonia varied significantly during this period and major glacial changes resulted in periodic extinctions of fauna associated with these near-shore ecosystems, allowing the colonization of vacant niches and creating opportunities for geographical isolation and speciation (Valdovinos et al., 2003; Zemlak et al., 2010; González-Wevar et al., 2011; Fraser et al., 2012; González-Wevar et al., 2012; González-Wevar et al., 2016; González-Wevar et al., 2018; Fernández-Iriarte et al., 2020). The Expansion-Contraction (E-C) model of Quaternary biogeography (Provan and Bennett, 2008) proposes that species and populations contracted their distributions to glacial refugia during glacial maxima. During interglacial periods they expanded their distributions towards previously glaciated areas following the deglaciation process (Provan and Bennett, 2008; Marko et al., 2010). Accordingly, unglaciated areas are expected to harbour higher levels of genetic diversity than ice-impacted areas, or newly founded ones. In contrast, glaciated areas should exhibit evidence of recent postglacial demographic expansions including lower levels of genetic polymorphism with small numbers of haplotypes dominating disproportionally large areas because of recolonization processes (Marko, 2004; Maggs et al., 2008). Moreover, recolonized areas should exhibit low divergence among haplotypes and lower levels of genetic differentiation than refuges (Provan and Bennett, 2008; Marko, 2004; Marko et al., 2010; González-Wevar et al., 2012; González-Wevar et al., 2013). This model provides a relatively simple paradigm to test demographic hypotheses during the Quaternary, and allows us to understand the response of populations and species to major climate change by recognizing distribution range shifts, potential refuge areas and recolonization routes (Hewitt, 2000, Hewitt, 2004; Maggs et al., 2008; Marko et al., 2010; Fraser et al., 2012; González-Wevar et al., 2013). *G. maculatus* includes migratory and landlocked populations distributed along the Pacific margin of South America between 30°S and the southern tip of Chilean Patagonia at 56°S. Phylogeographic and population-based analyses have recorded a small influence of Quaternary glacial cycles on the demography of the species (Zattara et al., 2005; Zemlak et al., 2010; Zemlak et al., 2011; González-Wevar. et al., 2015a; González-Wevar et al., 2015b). In general, *G. maculatus* is characterized by its high levels of genetic diversity and strong phylogeographic structure across its distribution (Zemlak et al., 2010; González-Wevar et al., 2015a; Delgado et al., 2019). Across non-glaciated areas between 38°S and 41°S each estuarine or riverine population represents a different genetic unit characterized by divergent haplogroups (genetic pool derived from shared mtDNA haplotypes). The diversity of *G. maculatus* includes four main haplogroups, three of them in the ice-free zone. The fourth haplogroup is the one that dominates Patagonia (south of 42°S). In contrast, ice-impacted populations along the Pacific margin, between 43°S and 53°S, were all included in a single haplogroup, with lower levels of polymorphism and genetic structure (González-Wevar et al., 2015a). This phylogeographic pattern is consistent with the Expansion-Contraction (E-C) Quaternary biogeographic model (Provan and Bennett, 2008).

Previous studies have shown diverse patterns of genetic differentiation in this species. Carrea et al. (2012) examined spatial patterns of genetic and phenotypic variability of *G. maculatus* from postglacial lakes in north-western Argentinian Patagonia and recognized three different genetic clusters using microsatellite markers. Moreover, genetic analyses of migratory and landlocked populations across a latitudinal gradient recorded higher levels of genetic structure in northern Argentinian Patagonia than in southern Patagonia (Carrea et al., 2013). Northern Patagonian populations of *G. maculatus* could have survived Quaternary glacial cycles *in situ*, while higher latitude populations were probably eradicated by extensive ice-sheet advances and retreats. Recently, Delgado et al. (2019), using neutral and putatively adaptive SNP loci, found that migratory populations in estuarine zones of the Chilean coast are highly differentiated from their landlocked counterparts. Moreover, migratory populations showed higher levels of gene flow and absence of site fidelity, while landlocked populations showed evidence of different colonization events with relatively low genetic diversity and varying levels of gene flow. Victoriano et al. (2020), using mtDNA sequences, observed that *G. maculatus* showed higher levels of genetic diversity and structure than the species *G. platei,* in the same river basin on both sides of the Andes. This result was explained by the different migratory and non-migratory behaviour of the two species.

To gain a better understanding of the genetic consequences of the Quaternary glacial in *G. maculatus*, and corroborate the genetic diversity and structure observed in the species, we compared landlocked and migratory populations from western Patagonia in areas that were differentially affected by ice advances and retreats. The populations were evaluated using two types of molecular markers, mitochondrial DNA sequences and nuclear DNA microsatellites. Following the E-C Quaternary biogeographic model, non-glaciated areas are expected to exhibit higher levels of genetic diversity and structure than heavily ice-impacted ones, for both migratory and landlocked populations. Similarly, migratory estuarine populations are expected to harbour higher levels of genetic polymorphism associated with older demographic histories, while landlocked populations should exhibit lower levels of genetic diversity and recent population expansions probably derived from associated estuarine/riverine ecosystems.

## 2 Materials and Methods

### 2.1 Sampling, DNA Extraction and Amplification

**FIGURE 1:**
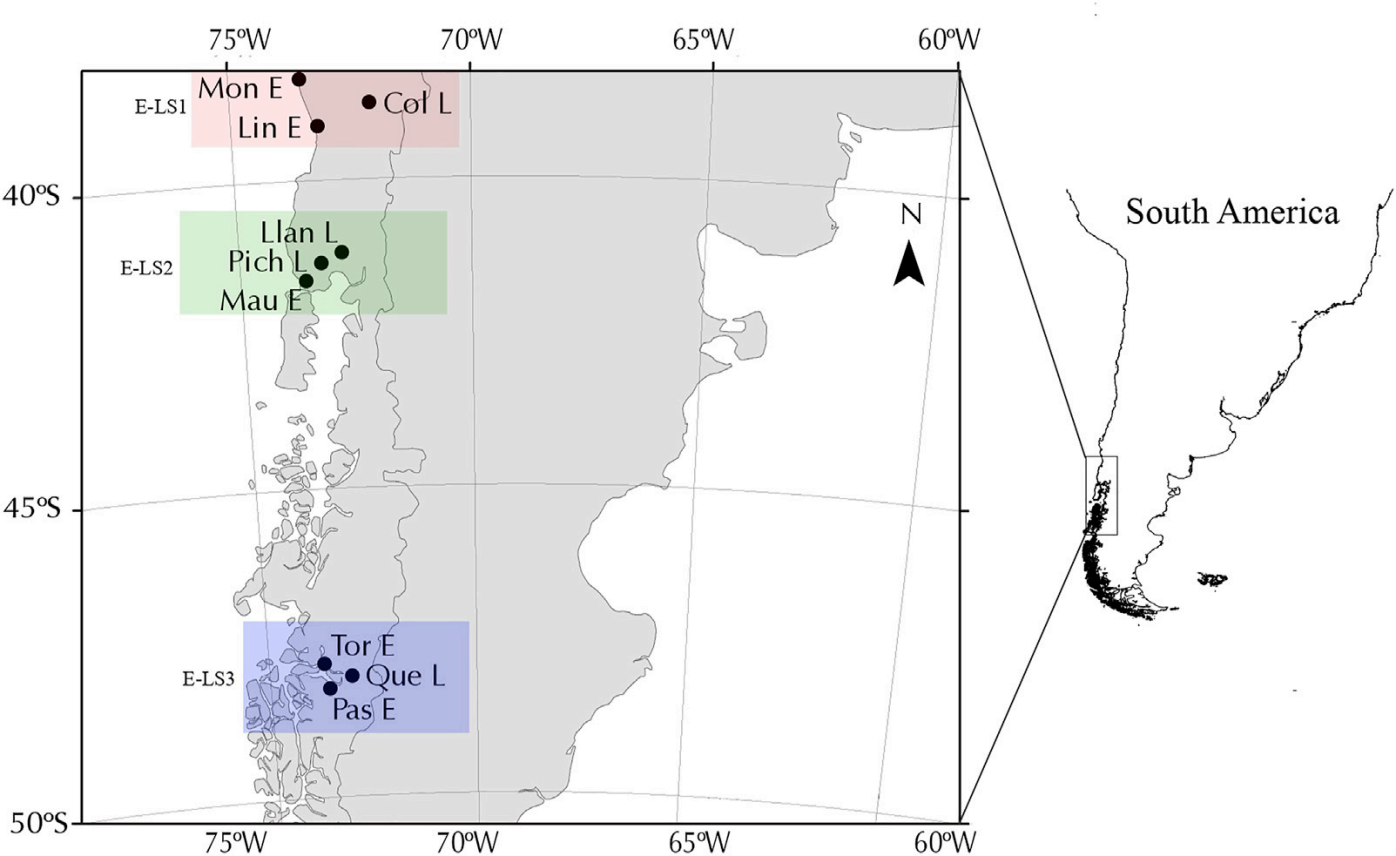
Map showing *Galaxias maculatus* sampling locations in western Patagonia separated into the three systems analysed.

Our analyses included nine populations of *G. maculatus* collected from lakes (*n* = 4) and associated estuaries (*n* = 5) in different biogeographical regions across the species distribution in southern Chile, in three areas that were differentially affected by Quaternary glacial processes (Figure 1). Firstly, we included two estuary-lake systems (E-LS) located in non-glaciated areas between 38°S and 42°S (Figure 1), also known as the Intermediate Area following Camus (2001). Localities in the first system (E-LS1) were Moncul Estuary (Mon-E); Lingue Estuary (Lin-E) and Colico Lake (Col-L) (Table 1). Localities in the second system (E-LS2) were: Maullín Estuary (Mau-E); Llanquihue Lake (Lla-L) and Pichilaguna Lake (Pic-L). We also included a third estuary-lake system located in an area that was heavily impacted by the Patagonian Ice Sheet in Magellan Province; localities in this third system (E-LS3) were: Tortel Estuary (Tor-R); Pascua Estuary (Pas-R); and Quetru Lake (Que-L) (Figure 1). Based on previous results (Gonzalez-Wevar et al., 2015a; Gonzalez-Wevar et al., 2015b), estuary populations are assumed to represent migratory populations and those located in lakes, landlocked ones. The geo-references of the sites are shown in Table 1 and their locations in Figure 1.

**TABLE 1:** *Galaxias maculatus* sampling sites in the estuaries and lakes of western Patagonia, separated into the three systems analysed, with sample size for molecular analysis.

System	Locality	Habitat	Code	Latitud	Longitud	N Mit	N Nucl
E-LS1	Moncul	Estuary	MON-E	38°42′S	73°24′W	24	25
	Lingue	Estuary	LIN-E	39°26′S	73°09′W	24	23
	Colico	Lake	COL-L	39°03′S	72°03′W	27	24
E-LS2	Maullín	Estuary	MAU-E	41°35′S	73°38′W	27	31
	Llanquihue	Lake	LLA-L	41°12′S	73°02′W	32	37
	Pichilaguna	Lake	PIC-L	41°16′S	73°03′W	29	19
E-LS3	Tortel	Estuary	TOR-E	47°45′S	73°32′W	27	27
	Pascua	Estuary	PAS-E	48°15′S	73°18′W	45	25
	Quetru	Lake	QUE-L	48°11′S	73°13′W	31	30

About 300 individuals were sampled during the year 2013, varying in a range of 30–50 individuals per locality, using whitebait nets and storage in ethanol (95%) for DNA preservation.

DNA was extracted from muscle tissue using 1) the salting-out method described by Aljanabi and Martinez (1997), which was applied to the samples analysed in the Genomics and Molecular Ecology Lab in Valdivia; or 2) the standard DNA extraction protocol with ethanol precipitation (Taggart et al., 1992), applied to the samples analysed in the Molecular Genetic Lab in Puerto Montt. Both methods showed the same quality of DNA collection and were used in both markers. A partial fragment of the mitochondrial Control Region (D-loop) was amplified using the specific primers GAL-F and GAL-R designed from the complete mitochondrial genome of the species (González-Wevar et al., 2015b). Nine microsatellite loci were amplified using specific primers developed by Carrea et al. (2009). PCR conditions for mtDNA and microsatellite amplifications were defined following González-Wevar et al., 2015a, Gonzalez-Wevar et al., 2015b and Carrea et al. (2009) respectively. Mitochondrial DNA amplification products were purified using QIAquick Gel Extraction Kit (QIAGEN) and sequenced in both directions with an Automatic Sequencer ABI3730 × 1 at Macrogen Inc. (Seoul, South Korea). The sizes of the amplified microsatellite alleles were determined using an automatic DNA sequencer (ABI Prism 377; Applied Biosystems).

### 2.2 Mitochondrial DNA Analysis

Mitochondrial sequences in *G. maculatus* were edited using GENEIOUS v.9.0.4 (http://www.geneious.com) and aligned with MUSCLE (Edgar, 2004). New D-loop sequences were deposited in GenBank under Accession Numbers: OM743508—OM743773. DNA saturation analysis was performed using DAMBE (Xia, 2013) to determine how saturation of transitions accumulates in relation to nucleotide divergence. Levels of genetic diversity of mtDNA marker were estimated through standard indices including: the number of haplotypes (*k*), the number of segregating sites (*S*), haplotype diversity (*H*), the average and number of pairwise differences (∏) for each locality, each estuary-lake system, and for the whole D-loop data set using DnaSP (Librado and Rozas 2009). We performed neutrality statistical tests (Tajima’s D and Fu’s FS) in DnaSP for each locality, each E-LS and the whole data set to estimate whether D-loop sequences in the species deviate from expectations under mutation-drift equilibrium. Considering the high levels of genetic diversity recorded in *G. maculatus* (Zemlak et al., 2010; González-Wevar CA. et al., 2015, Gonzalez-Wevar et al., 2015b, we determined levels of genetic differentiation between the localities analysed using mean pairwise differences (N_ST_) following Pons and Petit (1996) in ARLEQUIN v. 3.5 (Excoffier and Lischer, 2010). The statistical significance of these analyses was estimated through 20,000 permutations. We estimated phylogeographic structure using the nearest neighbour statistic (S_nn_), which measures how often nearest neighbours in sequences (in sequence space) are from the same locality in geographic space (Hudson, 1990). The statistical significance of S_nn_ was determined through 10,000 permutations. We determined the spatial genetic structure in *G. maculatus* through the number and composition of groups that were most differentiated based on mtDNA sequence data using SAMOVA (Spatial Analysis of Molecular Variance) (Dupanloup et al., 2002). SAMOVA is a popular analysis that uses multiple spatial scales in statistical methods to characterize spatial genetic structure based on pairwise genetic differences. We reconstructed mtDNA genealogical relationships in *G. maculatus* using Maximum Parsimony Networks in HAPVIEW (Salzburger et al., 2011). We also estimated the patterns of demographic history in the species by comparing the distribution of pairwise differences between haplotypes (mismatch distribution) for each locality, each E-LS and for the whole data set, up to the expected distribution under the sudden expansion growth model of Rogers and Harpending (1992) in DnaSP. Moreover, we estimated the mitochondrial age of the last demographic expansion in landlocked populations (Colico, Pichilaguna, and Quetru) under the sudden population growth model using the formula τ = 2 μt, where τ = the date of growth/decline measured in units of mutational time, t = time in years, and μ = the mutation rate per sequence per year. For this, we used a specific phylogeographic mutation rate estimated for the D-loop in galaxiid fish estimated by Burridge et al. (2008).

Finally, we performed phylogenetic reconstructions including the complete D-loop data set in *G. maculatus*, together with a sequence of *G. platei* as the outgroup. Bayesian reconstructions of haplotype relationships were done in MrBAYES v.3.1.2 (Huelsenbeck and Ronquist, 2001) using the GTR + I + G substitution model as previously selected with JModeltest v.2.1.10 (Darriba et al., 2012). Bayesian posterior probabilities (BPP) were estimated using the Metropolis coupled Markov-chain Monte-Carlo algorithm (MCMC). For this, we ran four chains for 150 × 10^6^ generations and trees were sampled every 1,000 generations. Stationarity of this analysis was inferred when the average standard deviation of split frequencies was lower than 0.01 as suggested by Huelsenbeck and Ronquist (2001). The first 5% of the parameter values were discarded as burn-in and posterior probabilities were calculated as the fraction of trees showing a particular node. Finally, posterior probability-density was summarized as a maximum clade credibility tree using TreeAnnotator v.1.6.1 (http://beast.bio.ed.ac.uk/TreeAnnotator) and edited using the FigTree v.1.4.3 programme (http://tree.bio.ed.ac. uk/software/figtree).

### 2.3 Microsatellite Analyses

Microsatellite fragment analysis was performed using Peak Scanner™ Software from Applied Biosystems and allele sizes were assigned to bins using FLEXIBIN (Amos et al., 2007). Finally, the evaluation of null alleles was reviewed and corrected using MICROCHECKER software v.2.2.3 (Van Oosterhout et al., 2004).

The statistical independence between loci was assessed using GENEPOP 4.7.5 (Rousset, 2008). Genotypic linkage disequilibrium between each pair of loci within populations was tested using Fisher’s exact test with a Markov chain. The deviation from genotypic proportion expected under the Hardy-Weinberg equilibrium was tested with exact *p* values by the Markov chain method using GENALEX 6.501 (Smouse and Peakall, 2012). Allele richness (Rs), standardized for the number of data, was calculated with FSTAT 2.9.4 (Goudet, 2002); the observed heterozygosity (Ho) and the expected heterozygosity (He) were estimated with GENALEX 6.501 (Smouse and Peakall, 2012). Microsatellite population genetic differentiation was assessed through F_ST_ comparisons between pairs of localities based on the total of polymorphic loci (Weir and Cockerham, 1984) using the sum of squared size differences (F_ST_-like) with *p*-value significance, implemented in ARLEQUIN 3.5 (Excoffier and Lischer, 2010).

We used different approaches to estimate the spatial distribution of genetic diversity using multilocus data. We established patterns of genetic differentiation among localities in *G. maculatus,* through Bayesian analysis implemented in STRUCTURE v.2.3.2 (Pritchard et al., 2000). To determine which K best fitted the data, we used ΔK methods described by Evanno et al. (2005); this method specifies that the most likely K is that found when this probability is plotted against successive values of K asymptotes, and does not increase significantly with increasing K. This analysis was estimated using the Structure Harvester web page (Earl and VonHoldt, 2012).

Finally, we performed a discriminant analysis of principal components (DAPC) using ADEGENET 3.1-1 (Jombart, 2008; Jombart and Ahmed, 2011) in the R platform (R Core Team, 2014). For this analysis, the number of clusters (K) was identified using the *find. clusters* function, which is based on the lowest values of the Bayesian information criterion (BIC). This type of analysis defines a model in which genetic variation is divided within and between groups, and which at the same time maximises the synthesis of the variables within groups and minimises it between groups using the information of the precedence of each sample as *a priori* information. Discriminant analysis (DA) achieves the best separation of the individuals within predefined groups and makes a probabilistic assignment of individuals to each group.

## 3 Results

### 3.1 Mitochondrial DNA

The complete mitochondrial DNA set in *G. maculatus* included 266 individuals collected from nine populations, with an average of 28 individuals per locality in a range from 19 to 45 individuals per locality (Table 2). The set consisted of 928 nucleotide positions. Considering that the analyses included a non-coding region of the mtDNA, several insertions and deletions were detected but not considered for future analyses. Control region sequences in *G. maculatus* were A—T rich (57.6%) compared to the G—C content (42.4%). Genetic diversity in *G. maculatus* was high with 197 variable positions (21.22%), of which 147 (74.61%) were parsimoniously informative. Levels of genetic diversity were much higher in migratory populations (estuaries) than in most of the landlocked populations (lakes), except for Llanquihue Lake, which exhibited high levels of polymorphism (Table 2). Haplotype diversity (H) ranged between 1.0 (Moncul, Maullín, Tortel and Pascua estuaries) and 0.729 (Quetru Lake) (Table 2). Similarly, the average numbers of nucleotide differences (Π) were higher in migratory than in landlocked populations, ranging from 30.33 (Maullín Estuary) to 1.72 (Colico Lake).

**TABLE 2:** Diversity indices and neutrality tests in *Galaxias maculatus* populations in the different E-LS across the species distribution in western Patagonia.

	D-Loop mitochondrial DNA						Microsatellites nuclear DNA			
Locality	K	H	S	Π	D	F_S_	Rs	Ho	He	F
MON-E	24	1.00	85	20.50	−0.76	−10.09*	10.62 ± 3.46	0.45 ± 0.07	0.78 ± 0.07	0.39 ± 0.10
LIN-E	18	0.94	77	22.77	−0.44	−0.61	10.56 ± 4.36	0.42 ± 0.04	0.76 ± 0.08	0.43 ± 0.06
COL-L	17	0.84	18	1.72	−2.29*	−14.48*	5.86 ± 3.67	0.45 ± 0.11	0.57 ± 0.09	0.25 ± 0.14
**E-LS1**	**59**	**0.97**	**103**	**20.75**	−**0.57**	−21.70*	**9.01 ± 4.34**	**0.44 ± 0.06**	**0.78 ± 0.06**	**0.42 ± 0.08**
MAU-E	27	1.00	109	30.33	−0.22	−9.18*	9.68 ± 3.39	0.34 ± 0.06	0.71 ± 0.08	0.47 ± 0.11
LLA-L	28	0.99	86	11.65	−1.91*	−13.31*	9.23 ± 3.79	0.59 ± 0.06	0.75 ± 0.04	0.20 ± 0.09
PIC-L	10	0.77	19	3.23	−1.15	−1.26	4.67 ± 2.84	0.57 ± 0.13	0.50 ± 0.09	−0.09 ± 0.18
**E-LS2**	**65**	**0.98**	**162**	**36.63**	−**0.14**	−12.53*	**7.86 ± 3.97**	**0.48 ± 0.05**	**0.79 ± 0.05**	**0.34 ± 0.10**
TOR-E	26	1.00	71	15.88	−1.01	−11.95*	10.37 ± 3.18	0.48 ± 0.06	0.81 ± 0.03	0.39 ± 0.07
PAS-E	43	1.00	75	13.24	−1.28	−32.40*	10.04 ± 3.48	0.42 ± 0.05	0.80 ± 0.05	0.48 ± 0.06
QUE-L	12	0.73	22	3.11	−1.60	−2.02	6.68 ± 2.40	0.49 ± 0.09	0.62 ± 0.08	0.27 ± 0.06
**E-LS3**	**81**	**0.98**	**94**	**14.38**	−**1.18**	−61.83*	**9.03 ± 3.39**	**0.46 ± 0.05**	**0.79 ± 0.04**	**0.42 ± 0.06**
Total	205	0.99	184	31.71	−0.45	−164.5*	11.70 ± 3.57	0.47 ± 0.03	0.70 ± 0.03	0.31 ± 0.04

Indices for D-loop from mitochondrial DNA where K, number of haplotypes; H, haplotype diversity; *S*, number of polymorphic sites; Π, average number of pairwise differences; D, Tajima’s D neutrality test and F_S_, Fu’s F_S_ neutrality test and indicators with standard error of the nine microsatellite loci; Rs, allele richness; Ho, observed heterozygosity; He, expected heterozygosity; F, inbreeding coefficient.

Global genetic indices for each E-LS are marked in bold. Statistical significance for Neutrality Test (Fs) are indicated with an asterisk.

Pairwise N_ST_ comparisons detected a high degree of genetic differentiation between migratory and landlocked populations of *G. maculatus* along a latitudinal gradient. All pairwise population comparisons with the exception of one (between Pascua and Tortel Estuaries), were statistically significant, showing the high degree of genetic differentiation reported in the species (Table 3). Across the sampling area, levels of mtDNA genetic differentiation were higher among lakes ( $\bar{X}$ = 0.947) than among estuaries ( $\bar{X}$ = 0.513), followed by the differentiation between lake and estuary populations ( $\bar{X}$ = 0.669). Specifically, migratory populations of the southern system (Tortel and Pascua Estuaries E-LS3) showed no significant genetic differentiation (Table 3). The nearest neighbour statistic S_nn_ in *G. maculatus* (S_nn_ = 0.815) showed a high and significant phylogeographic signal in the whole D-loop data set (*p* < 0.001). SAMOVA supported the general pattern of mtDNA structure and identified eight groups with maximum differences accounting for 66.09% of the total genetic variance, while only 0.06% of the variance was due to within-group variation among localities (Table 4). SAMOVA recognized each locality, except for Pascua and Tortel Estuaries (E-LS3), as different populations.

**TABLE 3:** Matrix of genetic differentiation values between pairs of *Galaxias maculatus* locations, estimated by mean general pairwise values of differentiation (N_ST_) of Mitochondrial DNA data (above diagonal) and estimated by F_ST_ obtained from the nine microsatellite loci (below diagonal).

	MON-E	LIN-E	COL-L	MAU-E	LLA-L	PIC-L	TOR-E	PAS-E	QUE-L
MON-E		**0.315**	**0.650**	**0.509**	**0.249**	**0.788**	**0.594**	**0.634**	**0.738**
LIN-E	0.014		**0.431**	**0.391**	**0.412**	**0.730**	**0.417**	**0.467**	**0.618**
COL-L	**0.216**	**0.446**		**0.683**	**0.750**	**0.949**	**0.807**	**0.805**	**0.944**
MAU-E	0.016	0.044	**0.146**		**0.583**	**0.711**	**0.289**	**0.355**	**0.519**
LLA-L	**0.100**	**0.124**	**0.264**	**0.139**		**0.848**	**0.664**	**0.686**	**0.796**
PIC-L	**0.270**	**0.333**	**0.460**	**0.296**	**0.135**		**0.810**	**0.812**	**0.936**
TOR-E	0.033	0.007	**0.430**	**0.077**	**0.135**	**0.386**		0.000	**0.463**
PAS-E	**0.057**	0.029	**0.350**	0.045	**0.186**	**0.288**	**0.101**		**0.473**
QUE-L	**0.086**	**0.051**	**0.507**	**0.156**	**0.123**	**0.399**	0.018	**0.161**	

Statistical significant pairwise comparisons are marked in bold.

**TABLE 4:** Spatial Analysis of Molecular Variance (SAMOVA) showing the percentage of genetic variation explained among groups (Moncul E, Lingue E, Colico L, Maullín E, Llanquihue L, Pichilaguna L, Quetru L, Pascua/Tortel E), among populations within groups and within populations using mtDNA. Where F_SC_ represents differentiation within populations among groups and F_CT_ represents differentiation among groups (***p < 0.001, **p < 0.01).

Source of variation	d.f.	Sum of squares	Variance component	Percentage of variation
Among groups	7	3067.993	13.34842 Va	66.09
Among populations within groups	1	7.237	0.01188 Vb	0.06
Within populations	257	1756.879	6.83611 Vc	33.85
Total	265	4832.109	20.19641	

Fixation index FSC: 0.00173*** FCT: 0.66093**.

Statistical significant pairwise comparisons are marked in bold.

**FIGURE 2:**
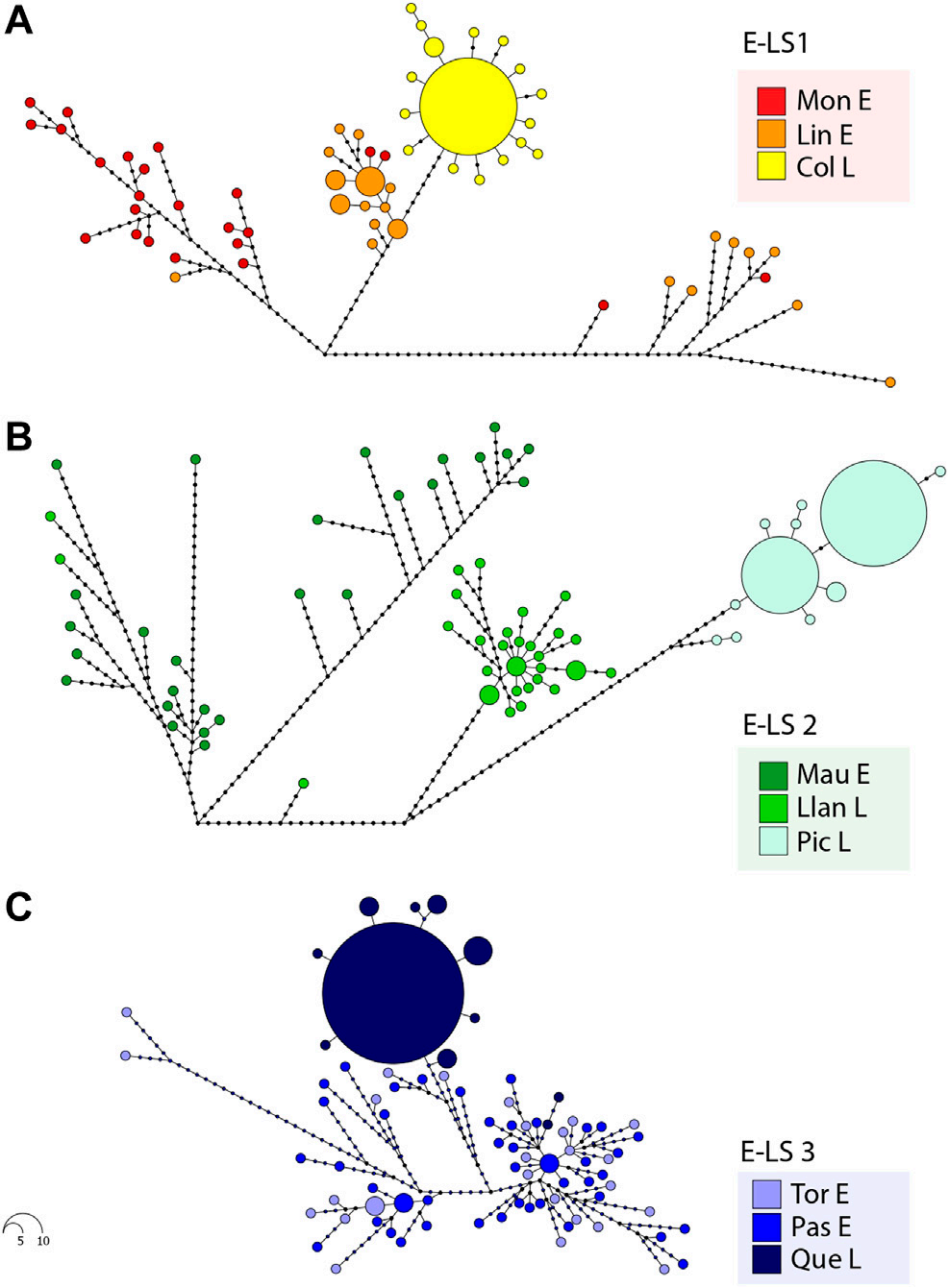
Maximum parsimony haplotype networks in *Galaxias maculatus* based on mtDNA D-loop sequences from: **(A)** Estuary-Lake System 1: E-LS 1 (Moncul Estuary: Mon E, Lingue Estuary: Lin E and Colico Lake: Col L); **(B)** Estuary-Lake System 2: E-LS 2(Maullín Estuary: Mau E, Llanquihue Lake: Llan L and Pichilaguna Lake: Pic L); and **(C)** Estuary-Lake System 3: E-LS3 (Tortel Estuary: Tor E, Pascua Estuary: Pas E and Quetru Lake: Que L). Each haplotype is represented by a coloured circle showing the locality where it was collected. The size of the circles is proportional to the frequency in the sampling effort.

Considering the levels of genetic diversity recorded in the species, haplotype network analyses were divided into the different estuary-lake systems (E-LS1—E-LS3) analysed here (Figures 2A–C). Maximum parsimony haplotype network in E-LS1 (Figure 2A) included a total of 59 different haplotypes, and Moncul Estuary showed the highest diversity with a total of 24 different haplotypes (H = 1.00). Lingue Estuary exhibited a total of 18 different haplotypes (H = 0.94), three of which were shared by more than one individual. Considering the expanded genealogies recorded at Moncul (Π = 20.50) and Lingue (Π = 22.77) estuaries, both localities exhibited multimodal distributions of pairwise differences between haplotypes. In contrast, the Colico Lake parsimony network included a total of 17 different haplotypes. A dominant haplotype was recorded in 12 individuals (44.4%), with several closely associated singletons (Figure 2A). Considering the short genealogy with a typical star-like topology recorded at Colico Lake, the distribution of pairwise differences between haplotypes presented a unimodal distribution (not shown). Maximum parsimony network in E-LS2 (Figure 2B) included a total of 65 haplotypes; Maullín Estuary showed the highest diversity with 27 different haplotypes (H = 1.00). Llanquihue Lake showed high levels of genetic polymorphism with 28 different haplotypes (*H* = 0.99), three of which were found in more than one individual. Considering the expanded genealogy recorded in Maullín Estuary (Π = 30.33) and Llanquihue Lake (Π = 11.65), both localities exhibited multimodal distributions of pairwise differences between haplotypes. Interestingly, two individuals collected in Llanquihue Lake are closely related to the diversity recorded in Maullín Estuary. In contrast, the parsimony network for Pichilaguna Lake included a total of only 10 haplotypes. Two dominant haplotypes were recorded in this locality, the first was found in 11 specimens (37.93%) and the second in 9 (31.39%). Another eight low-frequency haplotypes were closely associated with the dominant ones (Figure 2B). The pairwise differences in Pichilaguna Lake showed a bimodal distribution. The maximum parsimony network in E-LS3 (Figure 2C) included a total of 81 different haplotypes. Tortel and Pascua Estuaries showed the highest diversity with 26 and 43 different haplotypes respectively. Considering the expanded genealogy recorded at Tortel (Π = 15.88) and Pascua (Π = 13.24) Estuaries, both localities exhibited multimodal distributions of pairwise differences between haplotypes. In contrast, the parsimony network in Quetru Lake included a total of 12 different haplotypes. A dominant haplotype was recorded in 16 individuals (51.61%) surrounded by several singletons and low-frequency haplotypes. Interestingly, two individuals from Quetru Lake were closely associated with the diversity recorded in the associated tributaries (Pascua and Tortel Estuaries) (Figure 2C). Considering this, the distribution of pairwise differences between haplotypes from Quetru Lake exhibited a bimodal distribution. The age of population expansions estimated for landlocked populations of *G. maculatus* were 13,470 years for Colico lake, 16,840 years for Pichilaguna lake and 12,120 years for Quetru lake.

Phylogenetic reconstructions recognized three (HI, HIII and HIV) of the four main haplogroups previously recorded in *G. maculatus* in southern South America (Zemlak et al., 2010; González-Wevar et al., 2015a, Gonzalez-Wevar et al., 2015b. Haplogroup I was more abundant across the Intermediate Area localities while HIII was present in Lingue and Maullín estuaries (Supplementary Figure S1). Haplogroup IV was the dominant in estuarine and lacustrine populations located within the Patagonian Ice Sheet (Tortel Estuary, Pascua Estuary, and Quetru Lake) in areas that were heavily impacted by ice advances and retreats during glacial periods (Supplementary Figure S1). Some specimens from the localities of the Intermediate Area (Moncul Estuary, Lingue Estuary, Maullín Estuary and Llanquihue Lake) fell within the diversity of HIV, confirming the asymmetrical gene flow pattern (from north to south) previously found in the species (González-Wevar et al., 2015a).

### 3.2 Microsatellites

Genetic data for the microsatellite loci analysed were obtained from a total of 241 individuals collected from nine populations, of which 131 were collected in estuaries (migratory populations) and 110 in lakes (landlocked populations). Deviation from the Hardy-Weinberg equilibrium was observed in different loci combined with different populations (Supplementary Table S1). Excess homozygosity was observed, mainly through the presence of null alleles in some loci. Allele richness was higher in migratory localities (Rs = 10.25) than in landlocked populations (Rs = 6.61), with significant differences (*p* < 0.01) between the groups and when estuaries and lakes were compared within each E-LS (*p* < 0.01). In general, estuarine migratory populations showed greater allele richness than landlocked ones, except for Llanquihue Lake (E-LS2), which showed similar diversity to those recorded in migratory estuarine localities. The highest levels of allele richness were observed in migratory populations from Moncul and Lingue Estuaries (Table 2), and the lowest in landlocked populations including Pichilaguna, Colico and Quetru Lakes. The inbreeding coefficient (F; Table 2) showed no significant differences across a latitudinal gradient. When the genetic diversity was compared among systems (E-LS), no major differences were observed using microsatellites.

Overall comparison between locations showed a value of F_ST_ = 0.149. *A posteriori* pairwise analysis between locations based on the F_ST_ estimator (Table 3) showed significant differences between localities; this is explained mainly by the landlocked populations which differ between all the locations, except for Quetru Lake which showed no significant differences from Tortel Estuary.

Comparing only migratory populations (estuaries) with each other, we observed a low although significant genetic differentiation with F_ST_ = 0.058 (*p* < 0.001). When landlocked populations (lakes) were compared with each other, a higher genetic differentiation was observed (F_ST_ = 0.228), with significant differences (*p* < 0.001). When the estuaries were compared with the lakes within each system, the lowest genetic differentiation was observed between the localities of E-LS 3.

**FIGURE 3:**
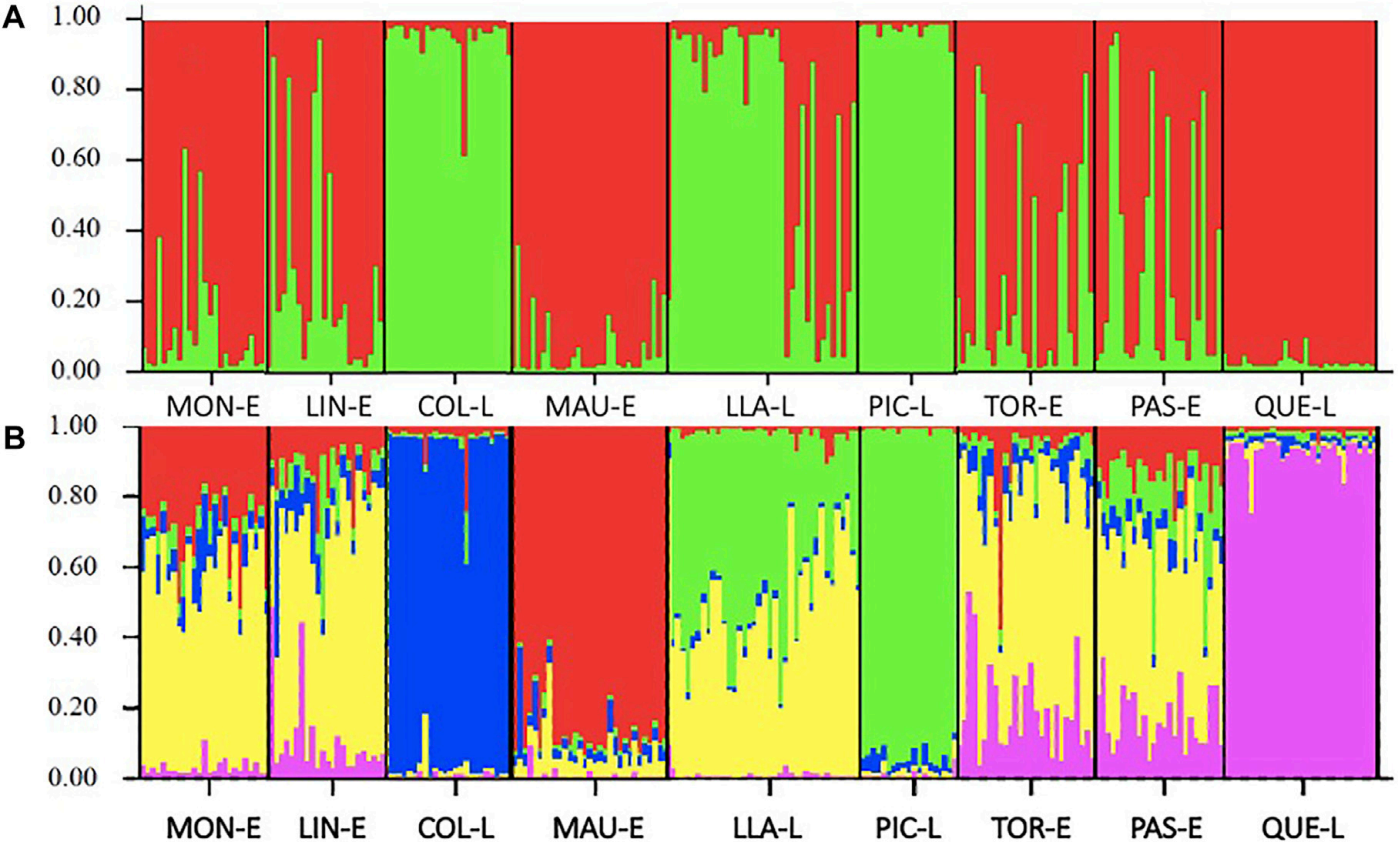
Genetic differentiation between locations, assessed by Bayesian analysis: **(A)** Diagram of the estimated population structure of Galaxias maculatus for the nine locations sampled with K = 2, the colours represent the genetic groups; **(B)** Diagram of population structure with K = 5. The name of the localities is observed on the lower horizontal axis: MON-E: Moncul Estuary; LIN-E: Lingue Estuary; COL-L: Colico Lake; MAU-E: Maullín Estuary; LLA-L: Llanquihue Lake; PIC-L Pichilaguna Lake; TOR-E: Tortel Estuary; PAS-E: Pascua Estuary and QUE-L Quetru Lake.

Bayesian analysis of genetic structuring in STRUCTURE showed two genetic groups differentiated from one another, represented by the colours distributed among the nine locations sampled (Figure 3) in which estuaries are mainly separated from lakes. The correlation proposed by Evanno et al. (2005) shows the point where ΔK reaches its maximum value, estimating that K = 2 (Ln P(K) = −9377.4) is the most probable K for the genetic groups identified in the sample (Figure 3A). Genetic differentiation was structured with two genetic clusters: one group with the individuals belonging to Colico, Llanquihue and Pichilaguna lakes and other cluster formed by all the rivers and Quetru Lake. However, when the results were observed with a higher value of K (K = 5) (Figure 3B), it was possible to identify a pattern of differentiation, consistent with all previous statistical results, in which high similarity among the estuaries except for Maullin Estuary and high differentiation between the lakes is observed, except for Lake Llanquihue.

**FIGURE 4:**
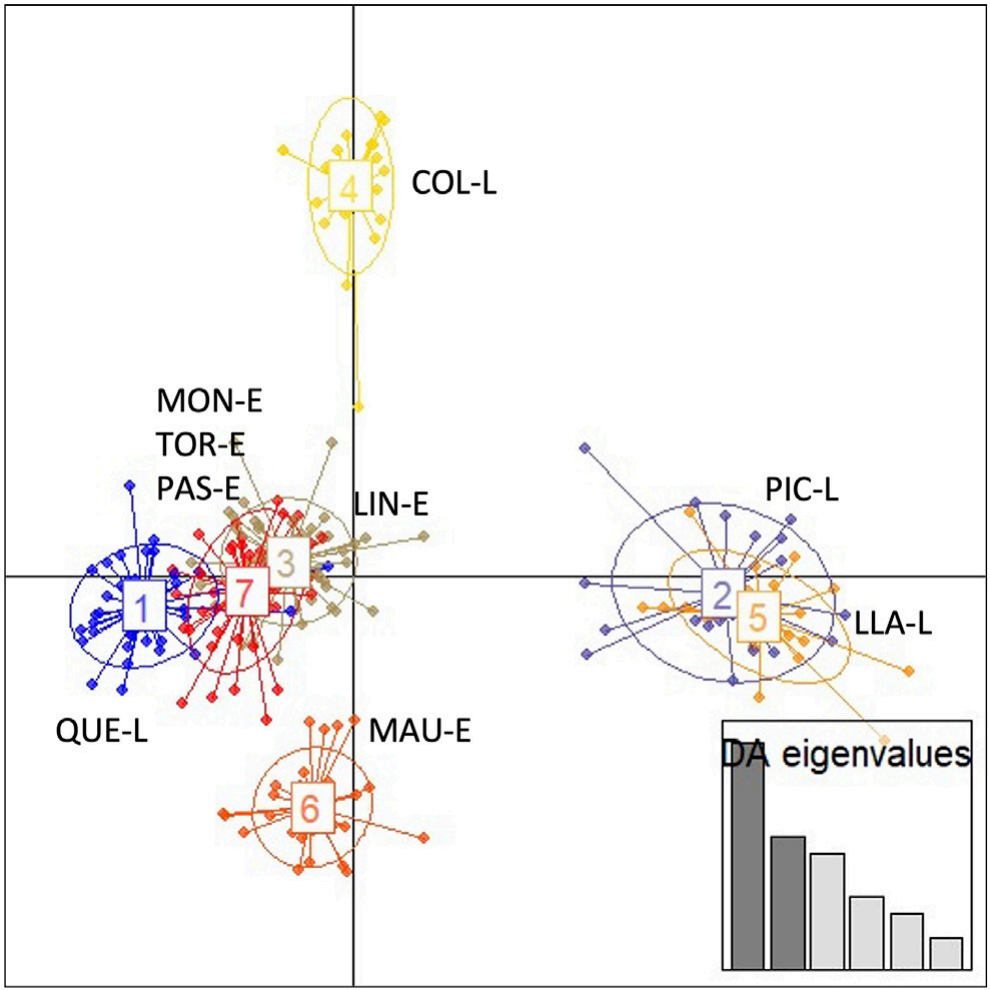
Ordering of the *Galaxias maculatus* clusters identified by the discriminant analysis of principal components (DAPC) of the different locations sampled. 1) Quetru Lake (QUE-L); 2: Pichilaguna Lake (PIC-L); 3: Lingue Estuary (LIN-E); 4: Colico Lake (COL-L); 5: Llanquihue Lake (LLA-L); 6: Maullín Estuary (MAU-E); 7: Moncul Estuary (MON-E)—Tortel Estuary (TOR-E)- Pascua Estuary (PAS-E).

The results of the discriminant analysis of principal components (DAPC) (Figure 4) showed a consistent pattern of genetic differentiation, similar to that observed in the Fst analysis. The highest differentiation was observed in Colico Lake and the lowest among estuarine localities. The results again group the individuals from Moncul, Lingue and Pascua Estuaries together as single groups, but close to Tortel Estuary. However, a clear separation is observed between estuary-lake systems. The individuals from Colico Lake clearly separate into a differentiated group, Llanquihue and Pichilaguna Lakes are seen to be segregated jointly, with these landlocked populations highly differentiated. The exception was Maullín Estuary, which is seen to form a more differentiated group.

## 4 Discussion

### 4.1 Genetic Diversity

#### 4.1.1 Mitochondrial DNA

As previously demonstrated in *Galaxias maculatus* (Zemlak et al., 2010; Zemlak et al., 2011; González-Wevar et al., 2015a; Gonzalez-Wevar et al., 2015b; Delgado et al., 2019), this species shows high levels of genetic diversity across its distribution in western South America. Among 266 individuals analysed with mtDNA, we recorded a total of 205 different haplotypes. On the one hand, levels of genetic diversity recorded in migratory populations of *G. maculatus* were higher across formerly unglaciated areas, while populations within the limits of the Patagonian Ice Sheet exhibited lower levels of polymorphism; accordingly, unglaciated areas represent more stable populations that were less affected by demographic processes associated with Quaternary glacial ice advances and retreats. On the other hand, the general diversity pattern recorded in migratory populations along a latitudinal gradient was not found in landlocked populations; levels of genetic diversity in landlocked populations of *G. maculatus* were lower and similar across our sampling effort, and probably a consequence of analogous demographic trajectories associated with recent bottlenecks and/or founder effects. Even though Colico and Pichilaguna lakes are located in unglaciated areas of western Patagonia, these lakes were covered by ice during the LGM (Moreno et al., 2018). Accordingly, the expansions of landlocked *G. maculatus* populations occurred after the Last Glacial Maximum, and hence are associated with post-glacial recolonization processes following the deglaciation process. As previously stated, the case of Llanquihue Lake is interesting and requires further study. Nevertheless, studies on *G. maculatus* have demonstrated a direct relationship between genetic diversity and the area of the lake analysed. Llanquihue is the second largest lake in South America; it is located at the limit of the expansion of glacial ice and probably served as a genetic reservoir for the species.

#### 4.1.2 Microsatellites

Microsatellite analysis in *G. maculatus* showed similar diversity patterns to those recorded in populations of the species from lakes and estuaries across Argentinian Patagonia (Carrea et al., 2012; Carrea et al., 2013). Our mean diversity and allele richness estimations are higher than those recorded by Carrea et al. (2013) in Argentinian Patagonia (Rs = 6.45). This study showed that migratory localities exhibited higher levels of genetic diversity than landlocked ones. It is to be expected that those populations with migratory behaviour will present greater genetic diversity than closed or landlocked populations, due to higher gene flow in the former and greater probability of genetic drift in the latter. Delgado et al. (2019) observe that estuarine migratory populations exhibited higher genetic diversity than resident populations, which is explained by the (probably low) diversity of the colonizing populations. Therefore, these differences in diversity between types of samples can be explained by bottlenecks generated by population reduction during glacial maxima, similar to those recorded by Zemlak et al. (2010) and Vera-Escalona et al., 2020.

### 4.2 Genetic Structure

#### 4.2.1 Mitochondrial DNA

As previously shown in this species (González-Wevar et al., 2015a), we also recorded contrasting patterns of genetic structure in migratory populations between the main biogeographic areas analysed. Each of the estuaries analysed north of the 42°S boundary, and across an area less than 250 km wide, represents a different genetic unit; the estuaries analysed in the southern system (E-LS3) of Patagonia exhibited lower levels of genetic differentiation. Again, major differences in terms of the genetic structure between unglaciated and glaciated areas are in basic agreement with the predictions of the Expansion-Contraction Quaternary biogeographic model in the south-eastern Pacific (Provan and Bennett, 2008; Pardo-Gandarillas et al., 2018; Fernández-Iriarte et al., 2020). González-Wevar CA. et al., 2015, González-Wevar et al., 2015b analysed populations from Chilean Patagonia and recorded low degrees of genetic structuring in estuary locations south of 42°S, belonging to the Magellan Biogeographical Province. These results coincide with observations in E-LS3 in this work, where the two estuaries showed no significant genetic differentiation. Thus, the genetic differences observed between the types of localities can be explained by the life cycles of the fish and their resident or migratory behaviour observed in the estuaries and rivers (Wallis et al., 2001; Delgado et al., 2019). Our results suggest a high probability of observing migratory behaviours in the estuary samples, and resident behaviours in lakes; the exception would be Maullín Estuary (MAU-E), where the slight genetic differentiation observed can be explained by the possible presence of a high proportion of individuals from resident populations.

Our results also show that the greatest differentiation is observed among all the landlocked populations. Quetru Lake, located in the area influenced by the LGM, showed the highest differentiation; it could have been affected by a process of extinction and recolonization from possible glacial refuges (Fraser et al., 2012).

#### 4.2.2 Microsatellites

Comparative analyses using microsatellites in *G. maculatus* detected significant differences among the populations analysed. However, pairwise analyses showed that these differences are mainly explained by the high genetic differentiation recorded in the landlocked populations, particularly those from Pichilaguna and Quetru Lakes. When landlocked and migratory populations were compared separately, we observed that the estuaries exhibit lower genetic structuring; in contrast, landlocked populations showed higher levels of genetic structure and fewer migrants. These observations were corroborated by the multivariate analyses (DAPC), where migratory populations from estuaries were grouped in a big central cloud; however, there are two clusters of higher similarity, one including Moncul, Lingue and Pascua and another one Tortel, all geographically distant from each another. These multivariate analyses showed that individuals sampled from landlocked populations could be separated from each other, and from migratory populations. Colico, Llanquihue and Pichilaguna Lakes showed the greatest similarity. Llanquihue and Pichilaguna Lakes are 3.5 km apart and are probably connected by streams. Llanquihue is the second-largest lake in South America, covering 860 km^2^; accordingly, it may represent a source for the *G. maculatus* population of Pichilaguna lake, which has an area of approximately 1 km^2^, although mtDNA data do not show shared genotypes. Zattara et al., 2005 found a positive correlation between genetic diversity, measured by isoenzymes, and lake area and perimeter; this agrees with the observations in our work, in which the lake with the largest area and perimeter (Llanquihue) has the highest allele richness, while the smallest lake (Pichilaguna) exhibited the lowest diversity of alleles. This is mainly due to the sizes reached by the populations in each lake; it is probable that populations of smaller effective size are more exposed to genetic drift or to the combined effects of bottlenecks, founder effects, endogamy, and restricted levels of gene flows, which would increase the inter-population genetic divergence in comparison with larger populations (Hartl and Clark, 1997). Just as was observed from the sequence data, Quetru Lake (E-LS3), located in the zone affected by the Last Glacial Maximum, shows greater differentiation and genetic structure than the lakes and estuaries located in the other two systems; this can be explained by the processes of extinction and recolonization in this area.

Finally, Bayesian Structure analysis showed a high differentiation among lakes and estuaries, separating *G. maculatus* populations into two main differentiated genetic groups. However, when evaluating this analysis with a greater number of differentiated groups (K = 5), it is possible to detect a differentiation pattern with high separation between lakes while the similarity between estuaries is maintained, which is consistent with our observations in the other analyses. The low differentiation detected among lakes by this Bayesian analysis may result from the high genetic diversity detected by microsatellite markers, due to their high mutational rate, which may generate allele similarity by homoplasies (Jarne and Lagoda, 1996; Estoup et al., 2002; Bhargava and Fuentes, 2010). The results obtained by the two types of markers are highly consistent; however, as observed in the structure result, a difference may be observed between the two molecular markers used. This could be due to the greater genetic diversity of microsatellites, which makes them very suitable for population analysis; however, in cases where the populations present high differentiation, it may be that the use of sequences as molecular markers can better detect the differentiation pattern than that shown by microsatellites. This would be due mainly to the possibility that the latter show high genetic similarity due to alleles that have already presented various mutations; in other words, for highly differentiated groups, the microsatellites could show homoplasies (Jarne and Lagoda, 1996; Estoup et al., 2002; Bhargava and Fuentes, 2010).

The different genetic statistical analyses show a consistent pattern of separation between the lakes analysed, and between lakes and estuaries. This pattern of genetic differentiation between lakes is similar to those recorded by Zattara et al., 2005, Delgado et al. (2019) and Rojo et al. (2020) using different types of molecular markers and analysing different watershed systems, and may be explained by the non-migratory behaviour of lacustrine populations. A similar pattern of genetic differentiation was recorded by Waters and Burridge (1999) when comparing mtDNA sequences from two Chilean lakes located in the same basin: Riñihue and Saval.

This high genetic differentiation, observed mainly in lakes and not in estuaries, may be because each lake is an “island”, and the separation between them acts as a barrier to gene flow. These results show that lakes behave as landlocked zones with little capacity for admixture, as there is probably little or no fish migration between lakes and estuaries. It has also been proposed that the level of structuring found in some basins (connected lakes, rivers, and estuaries) may be due to differential effects of Quaternary climate cycles in the different locations studied, since in some cases the population adopts a resident behaviour or life history, as observed in the variation to optional diadromous behaviour in a basin in Argentinian Patagonia (Carrea et al., 2013). This contrasts with the findings of Hollin and Schilling (1981) in locations at high latitudes which were strongly affected by glaciation in the mid-Pleistocene, the last glacial period when glaciers covered vast areas of Patagonia. This factor may have generated a refuge which now presents high genetic differentiation, as observed in the most southerly basin in Argentinian Patagonia analysed by Zemlak et al. (2008) and Carrea et al. (2013). In the present work, we observed a similar pattern of differentiation increasing with the latitude of the possible refuge zones affected by glaciation (Zemlak et al., 2008), mainly in Estuary-Lake System 3 (Tortel and Pascua Estuary with Quetru Lake), which would have been more strongly affected by the last glacial period than E-LS1 and E-LS2. However, a pattern of genetic differentiation was observed in the genetic diversity of E-LS3, explained by an effect of the last glacial period. In the case of lakes, which present high differentiation between one another and as compared to estuaries, the differentiation observed may be due to the possible presence of a refuge in each. In post-glaciation periods, these refuges would reconstruct highly differentiated populations. Thus, in the present work, using mtDNA sequences and microsatellite nuclear markers, we corroborated a pattern of differentiation between lakes and estuaries, similar to that already detected on a small scale by Zemlak et al. (2008) using mitochondrial DNA sequencing, and Delgado et al. (2019) using SNP markers. This corroborated the genetic separation of the lakes, which had already been evaluated through transplants by Delgado et al. (2020), where these differences come to be explained by evolutionary processes (natural selection or genetic drift); and by the presence of two types of behaviours in the same species, resident and migratory (Delgado et al., 2019). In addition, the effect of the Quaternary climate cycles on population structure and the loss of genetic diversity can be detected with both types of markers. Therefore, these patterns may be due to a combination of biological factors, i.e., resident non-migratory behaviour and/or landlocking and natal homing-in, as well as geological factors, i.e., E-C Quaternary biogeographic glaciation. It is therefore important to establish protection measures for this species, mainly in the lake populations due to the low diversity and high genetic differentiation observed in the lakes. Our findings need to be confirmed, to ensure the protection or conservation of these isolated populations.

## Data Availability

The datasets presented in this study can be found in online repositories. The names of the repository/repositories and accession number(s) can be found below: National Center for Biotechnology Information (NCBI) BioProject database under accession numbers OM743508—OM743773.

## Supplementary Material

Supplementary Figure S1 Bayesian maximum clade credibility tree of *Galaxias maculatus* haplotype relationships based on D-loop sequences. Bayesian posterior probabilities are shown above the nodes. Colored circles indicate sampling localities of the specimens analysed. Main haplogroups (HI, HIII, and HIV) recorded in *G. maculatus* are also indicated.

Supplementary Table S1 Deviation from the Hardy-Weinberg equilibrium in different microsatellites loci in different localities.
