# Supplementary material for: Contrasting Patterns of Genetic Diversity and Divergence Between Landlocked and Migratory Populations of Fish Galaxias maculatus, Evaluated Through Mitochondrial DNA Sequencing and Nuclear DNA Microsatellites
Source: Front Genet. 2022 May 19;13:854362. doi: 10.3389/fgene.2022.854362 (PMC9161745; doi:10.3389/fgene.2022.854362)
Supplement: Supplementary file 1 [file Table1.DOCX]

Table 1 Suplementary.

Summary of Chi-Square Tests for Hardy-Weinberg Equilibrium for each population in the 9 microsatellites loci.

|  | | | | | | |  | |  |
| --- | --- | --- | --- | --- | --- | --- | --- | --- | --- |
| **Pop** | **Locus** | **DF** | **ChiSq** | | **Prob** | | | **Signif** | |
| Pop1 | MG4 | 91 | 120,654 | | 0,020 | | | * | |
| Pop1 | MG2 | 36 | 75,844 | | 0,000 | | | *** | |
| Pop1 | MG5 | 78 | 118,186 | | 0,002 | | | ** | |
| Pop1 | MG9 | 10 | 4,143 | | 0,941 | | | ns | |
| Pop1 | MG6 | 120 | 163,334 | | 0,005 | | | ** | |
| Pop1 | MG8 | 66 | 70,815 | | 0,320 | | | ns | |
| Pop1 | MG3 | 136 | 320,153 | | 0,000 | | | *** | |
| Pop1 | MG7 | 28 | 156,907 | | 0,000 | | | *** | |
| Pop1 | G10 | 91 | 84,688 | | 0,666 | | | ns | |
| Pop2 | MG4 | 78 | 88,267 | | 0,200 | | | ns | |
| Pop2 | MG2 | 3 | 23,432 | | 0,000 | | | *** | |
| Pop2 | MG5 | 21 | 13,578 | | 0,887 | | | ns | |
| Pop2 | MG9 | 10 | 17,811 | | 0,058 | | | ns | |
| Pop2 | MG6 | 78 | 118,067 | | 0,002 | | | ** | |
| Pop2 | MG8 | 21 | 61,550 | | 0,000 | | | *** | |
| Pop2 | MG3 | 10 | 7,578 | | 0,670 | | | ns | |
| Pop2 | MG7 | 6 | 72,000 | | 0,000 | | | *** | |
| Pop2 | G10 | Monomorphic | |  | |  | | |  |
| Pop3 | MG4 | 45 | 41,993 | | 0,600 | | | ns | |
| Pop3 | MG2 | 28 | 91,530 | | 0,000 | | | *** | |
| Pop3 | MG5 | 45 | 101,669 | | 0,000 | | | *** | |
| Pop3 | MG9 | 3 | 3,011 | | 0,390 | | | ns | |
| Pop3 | MG6 | 105 | 179,080 | | 0,000 | | | *** | |
| Pop3 | MG8 | 55 | 120,372 | | 0,000 | | | *** | |
| Pop3 | MG3 | 190 | 259,747 | | 0,001 | | | *** | |
| Pop3 | MG7 | 66 | 161,333 | | 0,000 | | | *** | |
| Pop3 | G10 | 105 | 177,762 | | 0,000 | | | *** | |
| Pop4 | MG4 | 120 | 108,205 | | 0,772 | | | ns | |
| Pop4 | MG2 | 10 | 9,398 | | 0,495 | | | ns | |
| Pop4 | MG5 | 28 | 22,801 | | 0,743 | | | ns | |
| Pop4 | MG9 | 66 | 100,589 | | 0,004 | | | ** | |
| Pop4 | MG6 | 210 | 304,738 | | 0,000 | | | *** | |
| Pop4 | MG8 | 45 | 102,433 | | 0,000 | | | *** | |
| Pop4 | MG3 | 91 | 132,923 | | 0,003 | | | ** | |
| Pop4 | MG7 | 10 | 8,941 | | 0,538 | | | ns | |
| Pop4 | G10 | 136 | 398,185 | | 0,000 | | | *** | |
| Pop5 | MG4 | 28 | 20,937 | | 0,828 | | | ns | |
| Pop5 | MG2 | 1 | 15,390 | | 0,000 | | | *** | |
| Pop5 | MG5 | 28 | 28,204 | | 0,454 | | | ns | |
| Pop5 | MG9 | 3 | 1,188 | | 0,756 | | | ns | |
| Pop5 | MG6 | Monomorphic | |  | |  | | |  |
| Pop5 | MG8 | 1 | 18,000 | | 0,000 | | | *** | |
| Pop5 | MG3 | 21 | 18,224 | | 0,635 | | | ns | |
| Pop5 | MG7 | 6 | 0,281 | | 1,000 | | | ns | |
| Pop5 | G10 | 28 | 12,420 | | 0,995 | | | ns | |
| Pop6 | MG4 | 66 | 92,737 | | 0,017 | | | * | |
| Pop6 | MG2 | 36 | 27,609 | | 0,841 | | | ns | |
| Pop6 | MG5 | 78 | 175,206 | | 0,000 | | | *** | |
| Pop6 | MG9 | 10 | 9,585 | | 0,478 | | | ns | |
| Pop6 | MG6 | 171 | 350,160 | | 0,000 | | | *** | |
| Pop6 | MG8 | 45 | 82,233 | | 0,001 | | | *** | |
| Pop6 | MG3 | 28 | 124,308 | | 0,000 | | | *** | |
| Pop6 | MG7 | 91 | 340,208 | | 0,000 | | | *** | |
| Pop6 | G10 | 105 | 267,437 | | 0,000 | | | *** | |
| Pop7 | MG4 | 45 | 35,181 | | 0,853 | | | ns | |
| Pop7 | MG2 | 45 | 78,368 | | 0,002 | | | ** | |
| Pop7 | MG5 | 55 | 103,785 | | 0,000 | | | *** | |
| Pop7 | MG9 | 36 | 44,220 | | 0,163 | | | ns | |
| Pop7 | MG6 | 153 | 240,340 | | 0,000 | | | *** | |
| Pop7 | MG8 | 55 | 59,701 | | 0,309 | | | ns | |
| Pop7 | MG3 | 171 | 203,000 | | 0,048 | | | * | |
| Pop7 | MG7 | 36 | 151,207 | | 0,000 | | | *** | |
| Pop7 | G10 | 36 | 75,404 | | 0,000 | | | *** | |
| Pop8 | MG4 | 45 | 94,444 | | 0,000 | | | *** | |
| Pop8 | MG2 | 55 | 110,680 | | 0,000 | | | *** | |
| Pop8 | MG5 | 91 | 93,226 | | 0,416 | | | ns | |
| Pop8 | MG9 | 15 | 32,638 | | 0,005 | | | ** | |
| Pop8 | MG6 | 136 | 228,147 | | 0,000 | | | *** | |
| Pop8 | MG8 | 36 | 47,454 | | 0,096 | | | ns | |
| Pop8 | MG3 | 78 | 171,682 | | 0,000 | | | *** | |
| Pop8 | MG7 | 10 | 25,200 | | 0,005 | | | ** | |
| Pop8 | G10 | 105 | 253,288 | | 0,000 | | | *** | |
| Pop9 | MG4 | 45 | 33,322 | | 0,901 | | | ns | |
| Pop9 | MG2 | 10 | 36,524 | | 0,000 | | | *** | |
| Pop9 | MG5 | 36 | 51,740 | | 0,043 | | | * | |
| Pop9 | MG9 | 10 | 14,948 | | 0,134 | | | ns | |
| Pop9 | MG6 | 66 | 109,446 | | 0,001 | | | *** | |
| Pop9 | MG8 | 45 | 26,374 | | 0,988 | | | ns | |
| Pop9 | MG3 | 15 | 70,313 | | 0,000 | | | *** | |
| Pop9 | MG7 | 6 | 17,969 | | 0,006 | | | ** | |
| Pop9 | G10 | 55 | 60,554 | | 0,282 | | | ns | |
|  |  |  |  | |  | | |  | |
| **Key: ns=not significant, * P<0.05, ** P<0.01, *** P<0.001** | | | | | | |  | |  |
