# Supplementary figures and images for: Contrasting Patterns of Genetic Diversity and Divergence Between Landlocked and Migratory Populations of Fish Galaxias maculatus, Evaluated Through Mitochondrial DNA Sequencing and Nuclear DNA Microsatellites
Source: Front Genet. 2022 May 19;13:854362. doi: 10.3389/fgene.2022.854362 (PMC9161745; doi:10.3389/fgene.2022.854362)

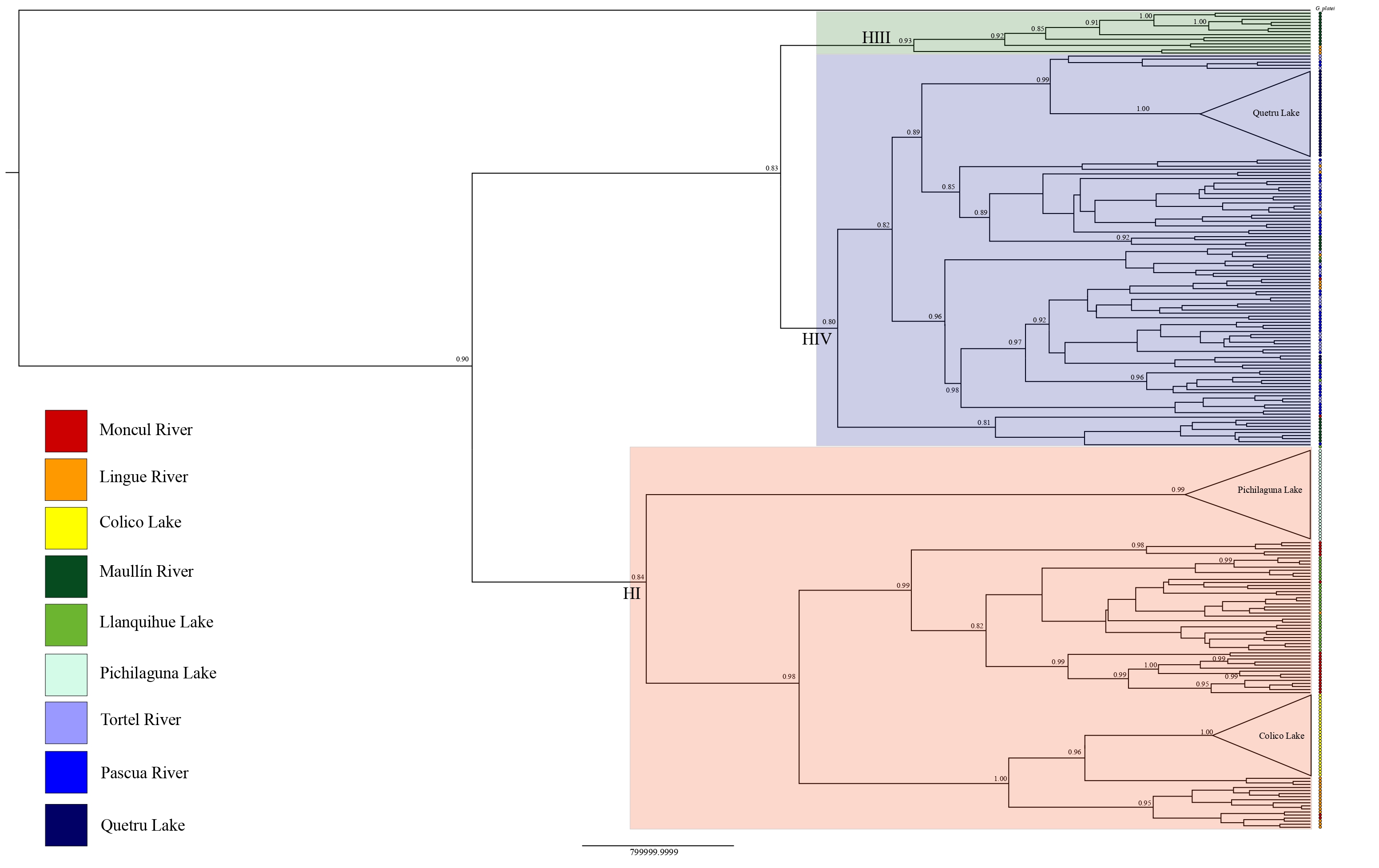

Supplement: Supplementary file 2 [file Image1.JPEG]
